# Exploring the consistency, quality and challenges in manual and automated coding of free-text diagnoses from hospital outpatient letters

**DOI:** 10.1371/journal.pone.0328108

**Published:** 2025-08-25

**Authors:** Warren Del-Pinto, George Demetriou, Meghna Jani, Rikesh Patel, Leanne Gray, Alex Bulcock, Niels Peek, Andrew S. Kanter, William G. Dixon, Goran Nenadic

**Affiliations:** 1 Department of Computer Science, University of Manchester, Manchester, United Kingdom; 2 Centre for Epidemiology Versus Arthritis, University of Manchester, Manchester, United Kingdom; 3 NIHR Manchester Biomedical Research Centre, University of Manchester, Manchester, United Kingdom; 4 The Northern Care Alliance NHS Foundation Trust, Salford, United Kingdom; 5 Manchester University NHS Foundation Trust, Manchester, United Kingdom; 6 University Hospitals of Morecambe Bay NHS Foundation Trust, Kendal, United Kingdom; 7 Division of Informatics, Imaging and Data Science, University of Manchester, Manchester, United Kingdom; 8 Manchester Academic Health Science Centre, Manchester, United Kingdom; 9 Intelligent Medical Objects, Inc., Northbrook, Illinois, United States of America; 10 Department of Biomedical Informatics and Epidemiology, Columbia University, New York, New York, United States of America; Public Library of Science, UNITED KINGDOM OF GREAT BRITAIN AND NORTHERN IRELAND

## Abstract

Clinical coding is the process of extracting key information contained within clinical free-text and representing this information using standardised clinical terminologies. In doing so, unstructured text is transformed into structured data that can be retrieved and analysed more effectively. This process is essential to improving direct care, supporting communication between clinicians and enabling clinical research. However, manual clinical coding is difficult and time consuming, motivating the development and use of natural language processing for automated coding. This work evaluates the quality and consistency of both manual and automated coding of diagnoses from hospital outpatient letters. Using 100 randomly selected letters, two human clinicians performed coding of diagnosis lists to SNOMED CT. Automated coding was also performed using IMO’s Concept Tagger. A gold standard was constructed by a panel of clinicians from a subset of the annotated diagnoses. This was used to evaluate the quality and consistency of manual and automated coding via (1) a distance-based metric, treating SNOMED CT as a graph, and (2) a qualitative metric agreed upon by the panel of clinicians. Correlation between the two metrics was also evaluated. Comparing human and computer-generated codes to the gold standard, the results indicate that humans slightly out-performed automated coding, while both performed notably better when there was only a single diagnosis contained in the free-text description. Automated coding was considered acceptable by the panel of clinicians in approximately 90% of cases.

## 1. Introduction

**Fig 1:**
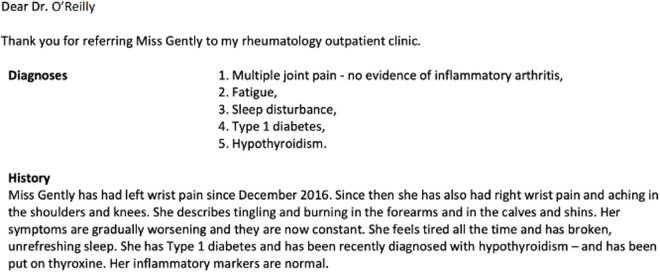
An example of an outpatient letter from the PRSB Outpatient Letter Standard. [1].

Healthcare is provided from a range of settings including general practice (GP), hospitals, pharmacies and care homes. Hospital outpatient departments provide specialist input to support the management of disease, advising patients and others in the healthcare system about diagnoses and management plans, often for long-term conditions. It is vital that documentation and communication are clear across different parts of the healthcare system, and that information is also conveyed clearly to patients. While some structured data is routinely collected, for example the occurrence of an outpatient visit in a particular speciality, further details of outpatient visits are often recorded solely as free text, such as in outpatient letters. The United Kingdom’s (UK) Professional Records Standards Body (PRSB) has developed guidance about what should be included in outpatient letters and how they should be structured, including headings such as demographics, referrer details, diagnoses, medications, history, plan and requested actions. Fig 1 provides an example letter, where free-text provides communication about the patient’s problems, including the clinician’s thinking about the patient’s diagnoses.

Although the primary purpose of collecting healthcare information is to support direct care and communication between healthcare professionals, there are well established secondary uses of healthcare data. These include improving the quality of patient care by enabling better planning, audit and quality improvement projects [2], as well as enabling research such as epidemiological studies [3]. In some countries, clinical coding is also frequently used for billing purposes [4]. Healthcare providers have therefore adopted the use of standardised clinical terminologies in many settings to support structured data capture [5]. This is common in some settings, for example GP surgeries, but is rare in others, such as outpatients, where free-text is often the only available source of data. However, free-text is not directly machine understandable, meaning that diagnoses and other information reported in outpatient letters cannot be viewed across a population of patients for any secondary use without additional processing. The lack of coded diagnosis data from hospital outpatient departments means, for example, that there is currently no national understanding of the distribution of diagnoses across patients in this setting, despite secondary care accounting for 72% of the annual National Health Service (NHS) commissioning budget as of 2016 in the UK [6]. Consequently, outpatient-based services and thus long-term conditions have a significant challenge as their data capture does not easily support secondary use of real-world data, and therefore progress and understanding of such conditions may be hampered. To mitigate that, national audits of certain long-term conditions have been set up to fill this gap. While such audits provide important findings [7], they often require bespoke data collection systems, duplicating data entry with significant additional time and resource, and also introducing possible transcription errors.

Mapping information within the text of an outpatient letter to a clinical terminology (often referred to as clinical coding) could provide the advantages of structured data capture, such as facilitating the interoperable storage, querying and exchange of clinical information among healthcare providers as well as population research. Depending on the provider and clinical setting, codes from different clinical terminologies may be used. Since 2018, the NHS has adopted the Systematized Nomenclature of Medicine Clinical Terms (SNOMED CT) terminology as a core terminology [5], as required by the Health and Social Care Act 2012. It is used to capture clinically relevant information such as diagnoses, procedures, symptoms, family history, allergies, assessment tools, observations, devices and other content to code care delivery to individuals.

The process of manual coding, either by clinical teams in real-time or by dedicated clinical coding teams, requires substantial training [8] and takes significant time as coding needs to be done for each individual patient and each encounter with the healthcare system. The transformation of large volumes of historical unstructured clinical documents, such as outpatient letters, into structured (coded) data presents an unfeasible manual challenge given the amount of unstructured information that exists within the healthcare domain and the available resources. Therefore, it is necessary to consider approaches to automatically map clinically relevant concepts from free-text to standardised clinical codes to alleviate this burden and unlock the potential of (decades of) information that is currently stored as unstructured data across the NHS by transforming it to structured, machine readable forms.

Text mining is a field of computer science that allows automated conversion of free-text information to structured, machine-understandable outputs [9,10]. The use of natural language processing (NLP) tools to extract and structure information from unstructured clinical text has been identified as one of the major areas of application for Artificial Intelligence in clinical care [11]. Automated coding via NLP techniques has the potential to make the task of coding clinical documents practical at a large scale, which has led to extensive exploration of the topic recently [12,13]. The task is often formulated as multi-label classification on either document or mention level, as several codes might be assigned to a given piece of clinical text. However, while recent neural models have had remarkable success in many healthcare applications, the accuracy of automated clinical coding is still relatively modest, oscillating around 60% [14–16]. Since clinical coding may not be perfect in all instances, it is important to understand how well both humans and text mining algorithms perform against a gold standard agreed upon by human clinicians. Currently there is not much work that evaluates the consistency of manual clinical coding, even within the same clinical settings [16]. In addition, existing gold-standard clinical datasets such as “Medical Information Mart for Intensive Care III” (MIMIC-III) [17] have been shown to be significantly under-coded [18]. The aim of this study was therefore to understand the comparability of manual and automated coding when converting free-text information about diagnoses to SNOMED CT codes using data from a dedicated diagnosis section within outpatient clinic letters, and to shed light to the coding differences observed between human coders and between the codes produced by automated software and those by human coders.

## 2. Methods and data

**Fig 2:**
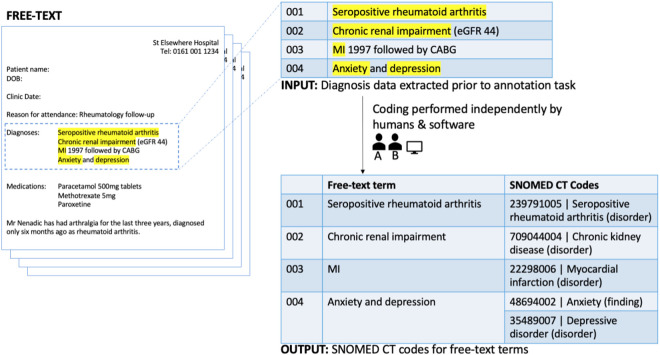
An illustration of the conceptual pipeline for mapping diagnosis descriptions.

In this paper we focus only on the semi-structured part of outpatient letters (see Fig 2), where a list of textual descriptions of diagnoses is provided. Each of these descriptions refers to one or more diagnoses and will be used as input for the clinical coding process. The main reason for using this list (as opposed to the narrative free text in the main body of the letter) is that we are interested in the quality and consistency of coding, both manual and automated, rather than in the assessment of both human and text mining capabilities to recognise and extract mentions of diagnoses in free-text narrative. Examining the quality of extracted diagnostic codes from the broader narrative in a letter, while important, lies outside the scope of this work. Nonetheless, insight gained from this work on the quality of coding of clinical diagnoses in text will also be informative for this broader task.

from free text to SNOMED CT codes. Note that for the text in line 004, it is necessary to make use of two separate SNOMED CT Clinical Finding codes. This is an example of a *code set* containing multiple codes.

The coding task was to map each of these individual lines of diagnosis descriptions to one or more relevant SNOMED CT concepts that encode the clinical intent (see Fig 2 and the task specification below). We selected SNOMED CT as the target terminology for this study, because it is focused on representing patient-level information at the point of care and has been adopted for this purpose by the NHS as a core terminology [5]. For the purposes of this work, the focus was on diagnoses. As such, the mapping was restricted to only those concepts that fall under the *Clinical Finding* subhierarchy of SNOMED CT. For example, in Fig 2, entry 001 would be mapped to a single code for “*Seropositive Rheumatoid Arthritis*”; in entry 004, there are two diagnoses to be coded: “*Anxiety*” and “*Depression*”, each with a separate SNOMED CT code. In this task, we did not consider procedures (e.g., “*Coronary artery bypass”*) or test results (e.g., the value of estimated glomerular filtration rate (*eGFR*)), or any other clinical concept types.

Given a single line with free-text description, the result of the task is a set of SNOMED CT codes (“code set”) that capture the clinical meaning, with respect to diagnoses, of the description. For example, given the free-text description “*Anxiety and depression*”, the coder, either human or computer, may return the result [48694002, 35489007] which are the SNOMED CT codes corresponding to “*Anxiety (finding)”* and *“Depressive disorder (disorder)”* respectively. We refer to this as the *code set* provided by the coder for the given free-text diagnosis. A code set may contain one or several SNOMED CT codes.

The overall methodology of the work presented here consists of the three steps: 1. Data acquisition and preparation; 2. SNOMED CT clinical coding; and 3. Coding evaluation. These are explained below in detail. Ethical approval for the project was obtained by IRAS (212818) and REC (16/HRA/4393). The data was accessed in April 2017. The authors had no access to information that could identify individual participants during or after data collection; all data was manually anonymised before the research team was given access. Participant consent was waived by the ethics committee as the data was fully anonymised.

### 2.1 Data acquisition and preparation

A random sample of 100 outpatient letters from 2013–2017 was retrieved from the Rheumatology department of Salford Royal Hospital, which is part of the Northern Care Alliance (NCA) NHS Foundation Trust, one of the largest NHS trusts in the UK. From these, a semi-structured list of diagnoses was manually extracted from each letter by the Digital team at the hospital, and shuffled randomly within the list, so that subsequent free-text lines were unlikely to belong to the same patient. The diagnosis descriptions were checked manually so that they contained no sensitive or identifiable information. Any descriptions that included irrelevant content, such as formatting notes (e.g., “----”) or empty lines, were excluded, resulting in a total of 708 free-text lines of diagnosis descriptions. The length of free-text diagnoses lines varied between 2 (e.g., “MI”) and 188 characters, excluding spaces, with a median length of 28 and an interquartile range of 26 (a lower quartile of 18 and upper of 44). Additional descriptive statistics are available in Appendix (A.1).

### 2.2 SNOMED clinical coding

#### 2.2.1 The task and coding guidelines.

The task was to code each line of textual diagnosis description to relevant SNOMED CT Clinical Findings, independently of other lines. With respect to the SNOMED CT ontology, we define *clinical findings* as any codes that are a descendent of “404684003 | Clinical Finding” in the SNOMED CT International Edition (as of Jan 2017). These codes include diseases (disorders), as well as other clinical findings such as symptoms (e.g., “*headache”*). The total number of such codes in the terminology was 107,465 as of Jan 2017. Although SNOMED CT includes the codes for other content to code care delivery to individuals, we decided to focus our investigations only on *clinical findings*, even if other information has been provided in free text and eventually coded by a coder. For example, given the following text:

*“Previous right knee meniscal repair with secondary osteoarthritis”*

the relevant information to be coded for this task was “*secondary osteoarthritis*”, which could be mapped to the SNOMED CT code “443524000 | Secondary osteoarthritis (disorder)”. However, the coding of other information including qualifiers, such as “*Previous*” and “*right*”, and procedures such as “*meniscal repair*”, was not necessary for the specified task. If these codes were included in the code set by a coder, then they were excluded from the analysis.

In collaboration with clinicians and coding experts, we developed a coding guideline (Appendix, A.5) that described the task and was used as a guide during the coding process. Specifically, the key coding principles included the following:

- The coding focus should be on clinical findings directly specified in a free-text diagnosis. No inference should be performed to derive or assume the existence of a diagnosis that is not directly mentioned or covered by the text. The only inference that should be performed during the coding process is the identification of an appropriate code for a disorder that is sufficiently described in the text, but uses different phrasing such as a synonym. For example, given *“scan showed Morton’s Neuroma”*, a coder may choose to code this as “30085007 | Morton’s metatarsalgia”, which is provided as a lexical synonym for “Morton’s neuroma” under the same clinical code 30085007 | Morton’s metatarsalgia (disorder). This choice is based on their own clinical judgement. If a free-text diagnosis mentions a clinical procedure or treatment, we do not infer the existence of a diagnosis unless the finding being diagnosed is explicitly stated in the wording of the procedure. For example, in “*cataract surgery*”, “*Cataract*” is coded as a disorder, whereas in “*eye surgery*”, there is no explicit mention of a disorder and therefore none should be coded.
- The chosen code should be as specific as possible so that it encodes the right clinical intent. If, however, the free-text description is unclear or underspecified, then a more generic code can be selected. For example, if a specific type of arthritis such as “*Psoriatic arthritis”* is not explicitly mentioned in the text, but might be (correctly or incorrectly) inferred by the clinician because of the presence of both “inflammatory arthritis” and “psoriasis”, then the more general parent concept “*inflammatory* a*rthritis”* should be used to annotate the text even though the more specific disorder could be inferred by the context.
- Pre-coordinated SNOMED CT terms should be used whenever possible. For example, “*severe asthma*” should be mapped to “370221004 | Severe asthma (disorder)”, rather than mapping it to two separate concepts: “24484000 | Severe (severity modifier) (qualifier value)” and “195967001 | Asthma (disorder)”. However, when coding a disorder for which there is not a single pre-coordinated code that fully captures the meaning of a clinical concept, the coder can add modifiers to the core concept, including locus/finding site, laterality, severity, chronicity/temporal associations, finding method and causative associations (such as coding “*infection*” in “*pancreatitis due to infection*”). This is often referred to as post-coordination. However, the focus of the analysis is still on identifying and coding core disorder concepts, while additional modifiers are used for clarity during the coding process.
- When two or more distinct clinical concepts are present in the same narrative description, these should be coded as separate code sets (recall entry 004 of Fig 2, where “*Anxiety* and *Depression”* should be mapped to two separate SNOMED CT concepts). Note however that this is not post-coordination: cases with multiple codes are separate set of codes, rather than a single post-coordinated concept. We refer to these as “multi-finding” cases, as opposed to those that contain “single-finding” only.

#### 2.2.2 Manual coding.

For each of the textual descriptions in the dataset, the **manual** coding was performed by two clinically-active clinicians, referred here as “coder A” and “coder B”. The coders were both practicing rheumatologists, with experience in using digital health technologies. The clinicians were asked to code each entry in the list of free-text diagnoses separately through the following steps:

- Identify core clinical findings in the free-text diagnosis, checking for multiple (distinct) clinical finding concepts appearing in a single free-text description.
- For each of these, use the SNOMED CT browser (http://browser.ihtsdotools.org/) to search for a pre-coordinated concept first, including trying synonyms, abbreviations or parent terms for the core concept. Once the core clinical findings are coded, the coder can post-coordinate the core concept with its qualifiers separately (although these qualifiers are excluded from the analyses we performed below).

Each coder performed the annotation task independently, providing a list of SNOMED CT codes for each line of free-text description. The list of 708 terms extracted from the letters was split in two subsets (for coders A and B). These two subsets had an overlap of 291 terms, which was coded by both coders. The coders were not aware of which terms were in the overlap. From this overlap set, a subset of 130 terms was used to create a gold standard (see below).

#### 2.2.3 Automated coding.

For **automated** coding, we used IMO Concept Tagger, a commercial software solution developed by Intelligent Medical Objects (IMO), which specializes in developing, managing and licensing medical vocabularies. Specifically, IMO’s clinical interface terminology maps diagnostic terminologies to SNOMED CT concepts by providing a bridge between terms used in clinical practice and standardized vocabularies. For each line of free-text diagnosis description, the text was sent to the software, which returned a coding of the text mapped to SNOMED CT. As with manual coding, the software only focused on the “Problem” codes, which correspond to SNOMED CT’s “Clinical Finding”.

#### 2.2.4 Gold standard.

Following the code sets produced independently by each human coder, additional sessions to create an agreed gold standard were organised with a panel of four clinicians (the two original coders and two additional clinicians) and one independent assessor. The four clinicians were practicing rheumatologists, where the independent assessor was a general physician external to Salford Royal hospital. To perform the task, the panel had access to the SNOMED CT browser and to the codes provided by the coders A and B. Each of the free-text descriptions was discussed by the panel to produce a gold standard code set. If the panel members were not in agreement, the independent assessor adjudicated.

The gold standard was created from a subset of 130 clinical diagnosis text descriptions. Of these, 27 descriptions were judged to refer to concepts that were not clinical findings corresponding to a diagnosis (e.g., they may refer to procedures), or were too vague to be coded (e.g., “Allergy”). Since this work focuses on coding of diagnoses, these cases were excluded. An additional one description was found to be included twice in the dataset by mistake. Consequently, the gold standard set refers to the remaining 102 diagnoses.

### 2.3 Coding evaluation and metrics

Three sets of comparisons were performed over the code sets produced: i) a human-to-human comparison, ii) a human to gold standard comparison, and iii) a computer to gold standard comparison. The human-to-human case compares the code sets provided by coders A and B. The human-to-gold standard case compares the codes provided by coders A and B to the gold standard provided by the panel of clinicians, where the code sets provided by each individual coder for a given diagnosis description are separately compared to the corresponding gold standard. The software to gold standard case compares the codes generated by the IMO Concept Tagger (software) to the gold standard provided by the panel of clinicians.

In each of these comparisons, we performed two types of analysis: a distance-based evaluation and a qualitative analysis. When comparing between pairs of coders, we note that the number of examples compared may differ even when the comparison is made against the same dataset (e.g., the gold standard). This is because if one (or both) of the coders provided an invalid code set, for example one that did not contain any valid Clinical Finding codes, then the example would be excluded from the results. Therefore, depending on the pair of coders being compared, the final number of examples compared in the results may differ (see Table 8 in the Appendix).

#### 2.3.1 Distance-based evaluation.

We used the SNOMED CT hierarchy to evaluate the similarity between two code sets that have been provided for a given free-text diagnosis (e.g., one by a human coder and one provided as a gold standard). When each of the code sets contains a single code only, then the similarity can be calculated as the minimum distance (i.e., shortest path) between the two codes: the shorter the minimum distance, the more similar the two codes are. If any of the code sets being compared have more than a single code, then we must use a metric that calculates a distance between two *sets* of SNOMED CT codes [19]. As we here consider diagnosis descriptions which may refer to multiple distinct clinical findings (“multi-findings” as mentioned above), the distance metric should only compare the corresponding codes in each code set that feasibly refer to the same finding in the diagnosis description, rather than “penalizing” the coding based on the diversity of findings described in the original text. To this end, we have defined the similarity as the average minimum distance between each code and its closest code in the other dataset. The full description of the distance-based evaluation can be found in Appendix (A.2). As an example, consider the following two code sets (derived for *“Left shoulder tendonitis and a fractured clavicle”*):

*Set 1 = {202852009 | Shoulder tendinitis; 58150001 | Fracture of clavicle}*
*Set 2 = {76318008 | Disorder of tendon of shoulder region; 58150001 | Fracture of clavicle}*

For the code “202852009 | Shoulder tendinitis”, the closest corresponding code in Set 2 is “76318008 | Disorder of tendon of shoulder region”. The distance between these codes is 2, which is calculated by following the path between the two codes, which is as follows:

*76318008 | Disorder of tendon of shoulder region*

*parent_of*

*239955008 | Tendinitis AND/OR tenosynovitis of the shoulder region*

*parent_of*

*202852009 | Shoulder tendinitis*

where “*parent_of*” refers to a “type of” (subsumption) relationship in SNOMED CT. For the other code in Set 1 (58150001), there is an exact match in Set 2, resulting in a distance of 0. Therefore, the average minimum distance metric would return 1 when comparing these two code sets.

Given the complexity of evaluating code sets containing multiple Clinical Finding codes, we stratify the free-text descriptions into cases with only one Clinical Finding code (“single-finding” cases for which both coders provided a single *Clinical Finding* code to annotate the given text), and cases in which the code sets contain multiple clinical finding codes (“multi-finding” cases). We also provide the results for “All” codes combined. Using the distance metric, we first analysed the number of exact matches, and then the number of matches that were within distance of 1, 2 or 3 or more of each other in the SNOMED CT hierarchy. We note again that we only focus on the *Clinical Finding* codes provided by each coder, and other codes are removed from the code sets.

#### 2.3.2 Qualitative analysis.

Using our panel of clinicians, we also performed a qualitative analysis of the assigned codes. This investigated to what extent the codes assigned by a human coder or the computer algorithm represented clinical intent expressed by free-text description, according to the judgement of the panel, in particular when there was not an exact match to the gold standard. The following ratings were provided when assessing the overall quality of the code sets provided by each coder:

- Good: the clinical judgement is that the assigned codes captured the clinical findings appropriately (to a high level). For example, given the text “*Seronegative psoriatic pattern arthropathy (plantar fasciitis, Achilles tendonitis)”,* an example of a “Good” coding is the following code set:
*399112009 | Seronegative arthritis (disorder),*
*202882003 | Plantar fasciitis (disorder),*
*11654001 | Achilles tendinitis (disorder)*
- Acceptable: the clinical judgement of the panel is that the code was relevant for capturing the main clinical intent for the diagnosis as a whole, although there might be some missing, broader/narrower, erroneous or inference-based codes in the set. For example, given the text *“Left side perineal tenosynovitis”,* an example of an “Acceptable” coding is:
*67801009 | Tenosynovitis (disorder)*

which is deemed to have a less specific (broader) meaning than the clinical notion described by the text.

- Not acceptable: the clinical judgement of the panel is that the code did not capture the clinical intent at an acceptable level. For example, given the text *“Mild colitis”,* the following is an example of an “Not acceptable” coding is:
*128524007 | Disorder of colon (disorder)*

as this concept is too broad to capture the specific meaning of the clinical notion in the text.

For each coder (human or software), we report the percentage of their codes that are considered “Good”, “Acceptable”, “Good” or “Acceptable”, and “Not acceptable”.

We also compared the results obtained using the distance metric to the qualitative analysis in order to determine the degree of correlation between the distance metric, based on the structure of the SNOMED CT ontology, and the qualitative evaluation provided by the expert panel. We first report the percentage of exact matches that were qualitatively categorised as “Good”, “Acceptable” and “Not acceptable”, and then we did the same for each of the distance of 1, 2 and 3 or more. Given that we are looking for a correlation between the distances and qualitative categories, we reported the results for all annotations together, rather than per individual coder.

## 3. Results

### 3.1. Distance-based evaluation

#### 3.1.1 Human to human agreement.

Table 1 shows the results of pairwise distance-based comparisons between the code sets provided by the human coders A and B (see also Table 9 in Appendix). Nearly all of the code sets provided by coder A and coder B fell within an average minimum distance of 3 from one another. Significant portions (73%) of these were exact matches.

**Table 1:** Distance-based comparison between code sets provided by the human coders (A/ B).

	Exact match (%)	Distance (%)		
		<=1	<=2	<=3
All	73	81	90	95
Single-finding	82	91	96	98
Multi-finding	8	17	42	75

The results also indicate that consensus was more likely to be reached in annotating text when the clinical meaning could be captured by a single Clinical Finding code, and conversely notably less likely to be reached when the meaning required multiple Clinical Finding codes to capture.

#### 3.1.2 Human to gold standard agreement.

The results in Table 2 shows the agreement between the two human coders and the gold standard (GS). The averages (Avg A/B) were obtained by micro-averaging.

**Table 2:** Distance-based comparison between the code sets provided by human coders (A and B) and the gold standard (GS).

		Exact match (%)	Distance (%)		
			<=1	<=2	<=3
All	Coder A vs GS	74	83	90	93
	Coder B vs GS	82	89	95	96
	Avg A/B	78	86	92	94
Single-finding	Coder A vs GS	86	92	96	98
	Coder B vs GS	89	94	98	98
	Avg A/B	88	93	97	98
Multi-finding	Coder A vs GS	0	23	46	62
	Coder B vs GS	40	60	80	87
	Avg A/B	21	43	64	75

The results show that in 78% of cases on average, the human coders matched the gold standard exactly, and that in 94% of cases the minimum average distance between corresponding codes in the human annotated set and the gold standard set was 3 or lower. Within the distance of 1 on single-finding cases, the agreement between the human coders and the gold standard was 93%. The correspondence between the clinicians’ codes and the gold standard was significantly lower for the multi-finding cases: an average of 21% of code sets provided exactly matched the gold standard, while 75% fell within an average minimum distance of 3 or lower from the corresponding gold standard code set.

#### 3.1.3 Computer to gold standard agreement.

Table 3 provides a distance-based comparison between the annotations produced by the software and the gold standard codes agreed upon by clinicians. The software gave an exact match in 62% of cases, 16 percentage points fewer than the clinicians. 12% fell outside the distance measure of 3 or more for the software, 6 percentage points more than for clinicians. However, within the distance of 1 in the single-finding cases, the agreement of the software with the gold standard (91%) was comparable to the same agreement level in case of the human coders (93%). Multi-finding cases performed less well, with 55% within a distance of three or less. Tables A.5 and A.6 in Appendix provide additional comparisons between the software and human coders.

**Table 3:** Distance-based comparison between the code sets provided by the software and the gold standard.

	Exact match (%)	Distance (%)		
		<=1	<=2	<=3
All	62	76	83	88
Single-finding	77	91	96	96
Multi-finding	0	15	30	55

### 3.2 Qualitative analyses

Table 4 provides the acceptability of codes provided by the human coders (A and B) and the software (Comp) as assessed by the clinical panel. On average, 85% of codes provided by the human coders fully capture the clinical intent (“Good”), with additional 12% providing an acceptable level. The acceptance of the software-generated code was around 10% lower for “Good” and for “Good” and “Acceptable” annotations.

**Table 4:** Capturing clinical intent for the human coders (qualitative analysis).

	Good %	Acceptable %	Cumulative (Good/ Acceptable) %	Not acceptable %
Coder A	83	15	98	2
Coder B	87	10	97	3
Avg A/B	85	12	98	2
Comp	75	14	88	12

### 3.3 Comparing the distance-based and qualitative metrics

**Fig 3:**
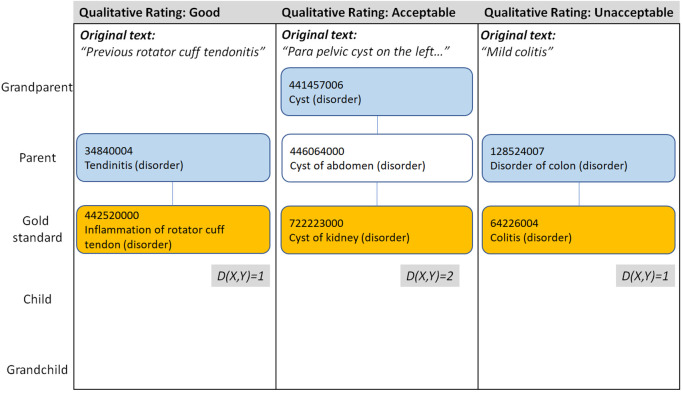
Examples of the qualitative and distance-based assessments of code sets. Example 1 (left): rated “Good” by the panel at a distance of 1 from the Gold Standard; Example 2 (middle): rated “Acceptable” by the panel at a distance of 2 from the Gold Standard; Example 3 (right): rated “Unacceptable” by the panel at a distance of 1 from the Gold Standard. Blue boxes indicate the codes provided by the coder, while yellow boxes represent the Gold Standard.

Table 5 compares the results obtained using the distance metric, where the distance is taken from each code set to the corresponding gold standard code set, to the qualitative analysis. Since the aim is to compare the two approaches to assessing the acceptability of a given code set, rather than the performance of the coders themselves, the results are given across all examples and are not grouped by individual coders as for the previous results. In 86% of cases when clinicians provided a rating of “Good” for a given code set, the codes used were an exact match to the gold standard codes. An additional 10% of “Good” cases were a distance of 1–3 from the gold standard codes (see Examples 1 and 2 in Fig 3). There were no exact matches in the “Acceptable” and “Not acceptable” groups. Less than half of the “Acceptable” codes were within a distance of 1 in the SNOMED CT hierarchy, while one in three were more than three steps away despite being Acceptable. Of the small number of “Not acceptable” code sets, 60% fell within a distance of 3 from the gold standard, with some even being within a distance of 1 (see Example 3 in Fig 3).

**Table 5:** Comparing the distance-based metric, from each provided code set to the corresponding gold standard, with the qualitative evaluation provided by the panel of clinicians.

	Exact match (%)	Distance to Gold Standard (%)		
		<=1	<=2	<=3
Good	86	91	94	96
Acceptable	0	43	60	69
Not Acceptable	0	20	53	60

## 4. Discussions

### 4.1 Summary of main findings

While there is a growing literature on clinical coding in general and to SNOMED CT in particular, there have been very few attempts to explore the consistency, quality and challenges in coding of real-world free-text diagnosis descriptions. The purpose of this study was to better understand these challenges and to shed light to the coding differences observed between human coders and between the codes selected by human and computer coders. The main findings can be summarised as follows:

- **Consistency of human annotations.** Human coders generally agreed on the clinical meaning of the presented text with respect to diagnoses, although they may have not always selected the same codes. The results indicated that clinicians agreed on exact codes for diagnoses contained in single line free-text descriptions in approximately 3 out of 4 cases, while nearly all of the code sets provided by human coders fell within an average minimum distance of 3 from one another.
- **Quality of coding.** Human coders agreed with or were close to the gold standard codes in the majority of cases, but found difficulty when the text contained multiple diagnoses, resulting in fewer exact or close matches to the gold standard for these cases. The software-generated coding had fewer matches to the gold standard than human coders, 62% exact matches as opposed to 78%, but were still close to the gold standard in the majority of cases in terms of distance in the SNOMED CT terminology. As with human coders, the performance of the software was lower over longer, more complex texts that contained multiple diagnoses.
- **Capturing clinical intent.** While there are inconsistencies and differences in both the human and automated coding compared to the gold standard, still 98% of human and 88% of automated codes were considered as “Good” or “Acceptable” in capturing the main clinical intent as assessed in the qualitative evaluation.

We also compared the qualitative evaluation to the automated distance metric. We found that the latter was a good proxy for the former: better qualitative ratings tended to correspond to code sets with a smaller distance from the gold standard codes. This indicates that the use of automated distance-based metrics utilising the structure of the ontology could serve as a lower-cost proxy to give an indication of the general quality of SNOMED CT codes assigned to free-text diagnosis descriptions. Nonetheless, in some cases, small distances were still considered to be “Not acceptable”, or large distances “Acceptable”. For example, example 3 in Fig 2 has a distance of 1 between the suggested and gold-standard codes, but was rated by the panel as “Not acceptable”. On the other hand, example 2 in Fig 2 has a greater distance of 2 between the suggested and gold-standard codes, but has been rated as “Acceptable”. This indicates a need for use-case specific evaluations of automated coding, and further development of metrics to evaluate both manual and automated coding in these cases.

We have further examined whether the “easy” and “difficult” examples (for both the distance-based and qualitative metrics) were the same for both human and software coders (for detailed results, see the Appendix, section A.4). In the case of distance-based comparison to the gold standard, in almost 60% of the examples for which a human coder provided the exact match to the gold standard, the software also provided the exact match. For almost all cases where the software provided an exact match to the gold standard, so did the human coders. On the other hand, in a small number of cases (around 6% on average), the software provided a coding that was more than three edges away from the gold standard, despite the fact that the human coder provided the exact match. This was the case mostly for multi-findings as the software either over- or under-coded. For example, in free-text description “STEMI (November 2012) - severe occlusion RAD stented, moderate LAD and moderate circumflex disease”, the human coders have chosen the gold standard code “401303003 | Acute ST segment elevation myocardial infarction (disorder)”), while the software – in addition to that code – also added the additional codes for “Reactive airway disease” (991000119106) and “Coronary artery stenosis (233970002)”, which has pushed the distance up (in part also because of the incorrect interpretation of RAD). This opens an interesting question as what should not be coded (e.g., because it is included in another code).

For the qualitative metric, the human coders received the same qualitative rating from the panel in 85% (87/102) of cases. On average in 75% (76/ 102) cases, the coding sets provided by both the human coder and the software were of the same quality according to the panel’s qualitative assessment. This means that in a quarter of cases, the level of complexity of free-text diagnoses was seen differently by the human and software coders. A third of such cases were cases for which the human achieved a rating of “Good”, while the software achieved a rating of “Not acceptable”. Conversely, there was only one example (example 3 in Fig 3, “Mild colitis”) for which a human coder received a rating of “Not acceptable” (as it was coded to “128524007 | Disorder of colon (disorder)”), while the software coding (“64226004 | Colitis (disorder)”) was rated as “Good”.

### 4.2 Implications for policy and practice

As clinical coding is a complex and challenging task for both human and automated coders, we note that there is a clear need to provide well-defined coding guidelines, as some inconsistencies in coding outcomes might be due to imprecise requirements, interpretation or restrictions during the coding (e.g., the use of pre- or post-coordinated terms). This also includes the need to situate annotation guidelines for specific tasks under a common framework [20] as well as incorporating principles and developments in representation formalisms for structuring clinical knowledge [21–24]. These considerations ensure that the results of manual or automated coding of clinical free-text are interoperable within healthcare systems and can be meaningfully compared to other annotated clinical text datasets.

The results in this work indicate that, in general, it is harder to perform manual or automated coding when diagnosis descriptions refer to multiple diagnoses, rather than a single one. Future guidelines for generating outpatient letters may be even more explicit in recommending the use of single clinical finding mentions within each line of a diagnosis list, given that they are more suitable for communication and easier to clinically code. Therefore, there is a need to improve automated coding over longer, more complex diagnosis texts. While these implications indicate potential directions for policy and practice, it is worth noting that in this work there were significantly more single-finding cases than multi-finding cases (7 times more).

### 4.3 Limitations

This work has several important limitations that need to be taken into account when interpreting the findings presented in this paper. Firstly, post-coordination was not considered for the coding of free-text diagnoses. As such, when mapping free-text to SNOMED CT codes, both human and software relied only upon pre-coordinated Clinical Finding codes. This is not a completely unrealistic setting in practice, as human coders in particular may want to focus on the most effective way to code by using pre-coordinated terms whenever possible. Still, this approach may present difficulty for the human coders who, depending on their experience with SNOMED CT, may rely upon the use of qualifiers to provide granularity to existing Clinical Finding codes. This is particularly true in the case where there is no suitable pre-coordinated Clinical Finding code available to capture the full meaning of the diagnoses occurring in a given text. Similarly, we note that the majority of automated clinical coding systems used to perform normalisation of free-text to SNOMED CT concepts do not include post-coordination. While systems may provide several codes for a given free-text description, they typically do not provide associations between disorder/finding terms and qualifiers. Post-coordination is however a strength of SNOMED CT: if coders have selected several codes for a given free-text description, it is possible to construct new expressions for meanings that are not captured by the terminology. Therefore, there is a need for annotation guidelines for both human coders and automated approaches to capture post-coordinated expressions from free-text, rather than rely on pre-coordinated codes only.

For this exercise, we created annotation guidelines (see Supplementary material) with instructions for coding to SNOMED CT Clinical Findings alone. The SNOMED CT vocabulary consists of various attributes including procedures, symptoms, morphological abnormalities, organisms, etc. Human coders sometimes mapped the given free-text diagnosis descriptions to SNOMED CT terms that matched closely to the original text, but did not include a Clinical Finding code, and these were considered as errors from the perspective of capturing diagnoses specifically. We also note that diagnosis descriptions were looked at in isolation (one at the time), without the complete clinical context; different coders might have considered this lack of context differently, which makes the individual codes somewhat subjective. While we looked at diagnoses extracted from outpatient letters, we also note that the context under which the free-text was originally produced will also influence the coding process: for example, a clinician may convey additional information about the primary diagnosis in free-text format, either as a note to themselves or to help the recipient of the letter. Furthermore, the coding process is also influenced by ‘local culture’, i.e., longstanding ways of working that prioritise codifying certain information and the functionality of systems such as the availability of an EHR and how long it has been used in the given department.

We also acknowledge that our qualitative categorisation is subjective: whether codes are “Good”, “Acceptable” or “Not acceptable” in practice will depend on the specific use case for the coded data, and will differ by context (e.g., direct care versus informing different types of population health research). For instance, finding all patients with a specific subtype of rheumatoid arthritis for a departmental query would require more specific codes, as opposed to finding anyone with any type of arthritis for a research study, where an exact match is less important. In other words, the same imperfect match may be considered ‘Good’ for one use case, and ‘Acceptable’ or even ‘Not acceptable’ for a different use case. Our qualitative evaluation was not linked to a specific context or task, and therefore the estimates provided in this paper need to be interpreted with caution. It may be feasible, and indeed useful, to conduct future evaluations in the context of particular use cases.

The data used in this case study were from a single discipline (rheumatology) and from a single hospital. While the dataset contained both rheumatological and non-rheumatological diagnoses for patients being seen in that clinic, the data would inevitably have been weighted towards diagnoses in this discipline and it is an open question whether the findings would be generalisable to other settings. Similarly, all the coders were rheumatologists. This has potential implications for deciding on the ‘gold standard’, and perhaps even more so for what was considered Good/ Acceptable/ Not acceptable. There may have been a disease-specific bias when allocating acceptability: for example, “*shoulder tendonitis”* was coded by computer to “76318008 | Disorder of tendon of shoulder region” but that was deemed too broad by the panel. However, a clinician performing the same exercise from a more general perspective, or with a different medical speciality that focuses less on rheumatological diagnoses, may consider the same result as acceptable.

## 5. Conclusions

In this work, we evaluated the quality and consistency of both manual and automated coding of diagnoses occurring in clinical text, specifically mapping diagnosis lists extracted from hospital OPLs to the SNOMED CT terminology. This evaluation considered both a distance-based metric, treating the SNOMED CT terminology as a graph, and a qualitative metric with a panel of clinicians to assess whether or not the provided codes captured the clinical meaning of the text. The findings indicate that human coders outperformed automated coding when compared to gold standard codes, though automated coding was still considered acceptable by a panel of clinicians in approximately 90% of cases. The performance of both human coders and automated coding was reduced when the diagnosis descriptions referred to multiple separate clinical findings, rather than a single main finding. Additionally, the distance-based metric calculated over SNOMED CT was found to be a good proxy for the manual qualitative evaluation.

## Recommendations

The use of standardised clinical terminologies such as SNOMED CT to represent patients’ healthcare journeys is a key component in facilitating the primary and secondary use of clinical data: raw non-coded data (such as free-text narrative or images) are difficult to search and analyse. Manual coding of clinically relevant concepts represented in free text is not only time consuming, but also likely to be inconsistent. This difficulty is due in part to the importance of context in the medical setting: which information should be coded, and how the required information should be coded, depends on a multitude of factors including who the user or recipient of the coded data is and the purpose for which the data is to be used. For example, in clinical research settings, the type of information to be coded, and the level of granularity used for different subject areas, will likely differ from settings where data is used to directly inform patient care. These considerations need to be part of the overall embedding of coding of clinical free-text into healthcare systems.

The problem of mapping clinical free-text to SNOMED CT is an important one, particularly as the terminology reaches broader adoption by healthcare providers nationally [5] and internationally [25]. Additionally, SNOMED CT is increasingly used and integrated alongside other widely used terminologies such as the International Classification of Diseases (ICD) and the UK’s Dictionary of Medicines and Devices (dm + d) [26–28], with closer integration between ICD and SNOMED CT as an emerging focus [29]. It is therefore important to understand how NLP tools perform in practice with respect to the terminology structure and the meaning intended in the text by humans, expanding and improving upon existing evaluation metrics used within the NLP community that do not necessarily capture these aspects directly.

Finally, our findings demonstrate that there may be a need for further development and use of guidelines [30] when writing outpatient letters: textual descriptions that refer to a single disorder are notably easier to code by both human and automated coders, so future recommendations may suggest that outpatient letters should strive to have one diagnosis per line to facilitate more efficient automated coding.

## Future work

Further work is also required to explore how coding can be made more efficient in practice, including post-hoc coding (coding of existing diagnosis descriptions). Our findings demonstrate that textual descriptions that refer to a single disorder are notably easier to code by both human and automated coders, so future recommendations may suggest that outpatient letters should strive to have one diagnosis per line to facilitate both more efficient automated future coding.

Ongoing advancements in NLP [31] should be used to further improve clinical coding and should be subject to increasingly comprehensive evaluations extending upon existing metrics, including those proposed here. Further research should also be done to develop new NLP approaches that focus on the extraction of qualifiers, such as finding sites, disease severities and uncertainties, to enable clinical coding of full SNOMED CT expressions rather than only atomic concepts. In turn, this will enable the full use of the semantics of the SNOMED CT terminology, based on the Web Ontology Language (OWL2), for rich data querying and analysis that is of importance to clinical research.

Our analyses also demonstrated that the current clinical coding practices could provide outcomes that – although not ideal – could be used to support a number of tasks, including epidemiological research that could benefit from computer coding on a large scale. Still, we note that the evaluation of the quality and acceptability of coding needs to be placed in a specific context and scenario of a use case, rather than being considered irrespective of what the codes will be used for. We also acknowledge that such evaluations are naturally subjective and that further work is required to develop task-specific evaluation settings. The ability to provide clinical coding on a large scale (e.g., by using semi- or fully-automated software-generated codes) will transform future population health research by providing access to coded diagnoses from different hospital specialists, as opposed to access only to coded data from GPs. Nonetheless, we still need to explore to what extent such codes will be useful for research, or indeed in clinical care, and what challenges this approach would bring to specific settings, and how these need to be mitigated.

## Supporting information

S1 File Appendix. (DOCX)
