# Supplementary material for: Exploring the consistency, quality and challenges in manual and automated coding of free-text diagnoses from hospital outpatient letters
Source: PLoS One. 2025 Aug 25;20(8):e0328108. doi: 10.1371/journal.pone.0328108 (PMC12377573; doi:10.1371/journal.pone.0328108)
Supplement: S1 File — (DOCX) [file pone.0328108.s001.docx]

# Appendix

## A.1: Dataset descriptive statistics

Table 6: description of the dataset used in this paper.

| Number of free-text diagnosis descriptions | 708 |
| --- | --- |
| Mean length (characters) | 36 |
| Min length of description (characters) | 2 |
| Max length of description (characters) | 188 |
| St. dev of length (characters) | 27 |

Table 7: the number of examples that have been coded manually by each of the coders (A and B). A subset of 291 examples were coded by both coders (independently). The entire set of 708 examples was coded by the software.

|  | Number of diagnosis coded |
| --- | --- |
| Coded by both A and B | 291 |
| Coded by coder A only | 191 |
| Coded by coder B only | 226 |
| Total terms manually coded | 708 |

## A.2: Distance-based comparison between coders

To evaluate the similarity between two code sets that have been provided for a given free-text diagnosis, it is necessary to define a suitable notion of similarity. Given the resource intensive nature of manual evaluation of code sets, it is beneficial to make use of a metric that can be automatically computed over the terminology from which the codes are derived. Additionally, since this work does not evaluate coding with respect to a specific application, it is necessary to define a generic metric that provides an indication of how similar two code sets are in general.

There are several important factors that should be taken into account by the metrics that are used to evaluate the similarity between the codes provided by each coder. In particular, the chosen metric:

- Should be able to account for sets of codes, rather than just single codes.
- Should not penalise based upon the number of codes used to annotate the given text, i.e., the evaluation should be consistent across sets of codes of varying sizes.
- Should not penalise based upon the diversity of codes used to annotate text, provided that there are similar codes across both of the sets being compared. It may be the case that a range of different diagnoses exist within the text and therefore similarity between two annotations should be based upon the presence of similar codes pointing to each separate diagnosis. Effectively, the metric should consider “subgroupings” of codes when measuring similarity.
- Should take into account “uncovered” codes, i.e., if one coder has identified a diagnosis and represented this by a code that is completely unrepresented in the set provided by the other coder.

Taking these points into consideration, the distance metric used for this evaluation is as follows:

$D\left( X,Y \right)=\frac{1}{|X\cup Y|}(\sum_{x\in X} {min}_{y\in Y}d\left( x,y \right)+\sum_{y\in Y} {min}_{x\in X}d\left( y,x \right))$

where X, Y are the sets of codes provided by each coder and *d(x, y)* denotes the minimum distance (shortest path) between codes *x* and *y*. Note that, given two code sets, for each individual code only the closest code in the other set is considered as part of the calculation. Effectively, the distance metric can be interpreted as the *average minimum distance* between each code and the closest code in the other code set.

Effectively, the metric attempts to group the codes in each set by the clinical concept they represent, calculating similarity based upon the closest match (minimum distances) rather than simply the distance to every other code in the other set.

It is worth noting that, if the metric D(X, Y) returns a value of 0, then this corresponds to the case in which the two sets contain exactly the same codes, i.e., the annotations provided by the two coders match exactly.

To perform the pre-processing and analysis, the January 2017 release of SNOMED CT International Edition was required in Web Ontology Language (OWL) format. The release format 2 (RF2) files for this release were converted to OWL format using the tool available at <https://github.com/IHTSDO/RF2-to-OWL>

This conversion was necessary to make use of the OWL API, and the description logic reasoner ELK, to reason over the SNOMED CT ontology. The implementation used was written in Java.

The implementation was used for the following:

- **Restricting the data to only the relevant clinical codes**. This was done by checking whether or not a given code was a descendent (subclass) of the *404684003 | Clinical finding (finding)* SNOMED CT concept using ELK. If a code was not a subclass of Clinical Finding, it was excluded during pre-processing. If a given annotation result did not contain any *Clinical Finding* codes, then the result was excluded from the analysis.
- **Calculating distance metrics for the comparisons of annotation results**. These distances were calculated by classifying the SNOMED CT ontology using ELK. Effectively, this provides a graph in which each node is a concept (code) and each edge is a subsumption (“is-A”, subclass) relationship between two codes. Distances between two codes can then be calculated by traversing the graph (calculating the distance to and from the least common ancestor of two codes). Over this graph, the *minimum distance* between two codes can then be defined as the shortest path between their corresponding nodes. This can be extended to code sets as given by the distance metric described earlier. Note that the graph includes implicit (as well as explicit) relationships between codes, due to the use of the OWL reasoner.

As an example of the pre-processing step, given the text *“Osteoarthritis - multi level degenerative changes*” the coder might provide the code set containing the codes 396275006 | Osteoarthritis (disorder)^[[1]](#footnote-1)^ and 33359002 | Degeneration (morphologic abnormality). Since the second code is not a *Clinical Finding* code, the code 3335902 is removed from the original result, where the resulting code set is then taken as the final coding for this description and coder for the purposes of the analysis. Similarly, for code sets containing codes that were erroneous, i.e., absent from the terminology (perhaps due to typographic errors while coding), then these were also removed during pre-processing.

The notion of distance is still applicable when comparing code sets of different sizes. For example, given the text “Previous right knee meniscal repair with secondary osteoarthritis” and the following code sets:
 *Set 1 = {239873007 | Osteoarthritis of knee; 443524000 | Secondary osteoarthritis}*

*Set 2 = {239873007 | Osteoarthritis of knee}*

The first code in code set 1 will be compared to the closest corresponding code in set 2, which is in this case an exact match (distance 0). The second code, “443524000 | Secondary osteoarthritis” will also be compared to the closest corresponding code in set 2, which in this case means it will also be compared to “239873007 | Osteoarthritis of knee”. The path between these two codes is via a common ancestor, “396275006 | Osteoarthritis” as follows:

443524000 | Secondary osteoarthritis

is_a

396275006 | Osteoarthritis

and

239873007 | Osteoarthritis of knee

is_a

396275006 | Osteoarthritis

resulting in a distance of 2. Therefore, the average minimum distance between the two code sets is 1. As such, the metric indirectly penalises the absence of “Secondary osteoarthritis” in the second code set. In some cases, this behaviour results in a larger distance (and hence “less ideal”) between two code sets when there is missing information in one set. However, there may be cases where a difference between the size of two code sets does not necessarily imply missing information: multiple codes may be used to express the same or similar information to a single code. Additionally, the importance of missing information depends upon what the information is and the application for which the code sets are being evaluated.

## A.3 Distance-based comparisons - additional results

When pairwise comparing a number of code-sets between two coders (two human coders, a human and software coder, or a coder and the gold standard), we note that the number of examples differed depending on which pair of coders was being compared. The reason for this is that a comparison could not be made if one or both of the coders did not provide Clinical Finding codes for a given example. The number of examples where the both coders have provided Clinical Finding codes for the same free-text description is used as a denominator in the corresponding calculations. Table 8 shows the numbers of examples used for specific pairwise comparisons.

Table 8: Number of examples (including single- and multi-findings) for each pairwise
comparison for which both coders provided a code set containing Clinical Finding codes.
Human coders A and B; Comp = software; GS = gold standard dataset.

| Pairwise  Comparison | Total examples compared | Single-finding  examples | Multi-finding examples |
| --- | --- | --- | --- |
| A vs B | 97 | 85 | 12 |
| A vs GS | 98 | 85 | 13 |
| B vs GS | 99 | 84 | 15 |
| Comp vs GS | 99 | 79 | 20 |

## Human to human agreement (larger dataset)

To make the pairwise comparisons comparable and consistent, all the results reported in the main text refer to the Gold Standard dataset (see below). This included the comparison between two human coders (Table 1), which was performed on the examples from the Gold Standard dataset. Since we had a larger dataset coded by both coders (see Table 6), we repeated the analysis on the subset of 291 double annotated examples. The results (Table 9) are consistent with the results obtained on the smaller dataset (Gold Standard).

Table 9: Comparison between human coders (A and B) - all results.

|  | Number of  instances | Exact  match (%) | Distance (%) | | |
| --- | --- | --- | --- | --- | --- |
|  |  |  | <=1 | <=2 | <=3 |
| A vs B (GS dataset) | 97 | 73 | 81 | 90 | 95 |
| A vs B  (larger dataset) | 227 | 73 | 80 | 89 | 93 |

We note that the comparison on the GS dataset was on 97 instances (out of the total of 130 in the GS dataset), whereas the larger analysis was on 227 instances (out of the total 291 in the dataset). See the previous paragraph for clarifications.

## Human to computer agreement

We also performed comparisons between the human coders and the software on both the Gold Standard and entire available (“larger”) datasets. We note these were direct pairwise comparisons, rather than the comparisons reported in Tables 2 and 3, which referred to the evaluation against the Gold Standard. The aim here was to understand if the human and automated coders would agree on specific instances, rather than whether the coding is correct.

The results on both the Gold Standard dataset (Table 10) and the larger dataset (Table 11) indicate that the average proportion of exact matches between the human clinicians and the software was around 62%, while for 88-90% of the code sets the average distance between the human and software annotated codes was 3 or less. This is almost exactly the same as the agreement between the Gold Standard codes and the software (Table 3). As expected, the agreement is notably better in the single-finding cases, where the exact matches between the codes provided by human coders and software were recorded in 75% of cases, and the distance of 3 or less in on average 96% of cases.

Table 10: Pairwise human to computer comparisons on the Gold Standard dataset.
The averages (e.g. Avg A/B) were obtained by micro-averaging.

|  |  | Exact  match (%) | Distance (%) | | |
| --- | --- | --- | --- | --- | --- |
|  |  |  | <=1 | <=2 | <=3 |
| All | A vs Comp | 64 | 79 | 85 | 90 |
|  | B vs Comp | 61 | 74 | 84 | 91 |
|  | Avg A/B | 62 | 77 | 85 | 90 |
| Single-finding | A vs Comp | 75 | 93 | 95 | 95 |
|  | B vs Comp | 74 | 90 | 96 | 96 |
|  | Avg A/B | 75 | 91 | 96 | 96 |
| Multi-finding | A vs Comp | 6 | 13 | 38 | 63 |
|  | B vs Comp | 10 | 15 | 35 | 70 |
|  | Avg A/B | 8 | 14 | 36 | 67 |

Table 11: Pairwise human to computer comparisons on the larger datasets. The averages (e.g. Avg A/B) were obtained by micro-averaging.

|  | Number of  instances | Exact  match (%) | Distance (%) | | |
| --- | --- | --- | --- | --- | --- |
|  |  |  | <=1 | <=2 | <=3 |
| A vs Comp | 347 | 62 | 73 | 82 | 88 |
| B vs Comp | 356 | 62 | 71 | 82 | 88 |
| Avg A/B |  | 62 | 72 | 82 | 88 |

# A.4 Coding similarity

While Section A.3 looked at the agreement between the coders even when the coding is not correct, we have further examined the level of agreement (or similarity) between the human coders and software agree with regards to the quality of coding examples. We focused only on the Gold Standard dataset and, for each free-text example, we first compared the distance between the provided codes and the Gold Standard. This has resulted in three similarity matrices (between coders A and B; coder A and software; coder B and software – see Table 12).

Table 12: Similarity matrices with distances D(X,Y) to the Gold Standard.
The tables show the numbers of cases rather than percentages.

|  |  | Coder B | | | | |
| --- | --- | --- | --- | --- | --- | --- |
|  |  | Exact | 0 < D(X, Y)  <= 1 | 1 < D(X, Y)  <= 2 | 2 < D(X, Y)  <= 3 | D(X, Y)  > 3 |
| Coder  A | Exact | 65 | 3 | 2 | 1 | 1 |
|  | 0 < D(X, Y) <= 1 | 3 | 3 | 2 | 0 | 0 |
|  | 1 < D(X, Y) <= 2 | 4 | 1 | 2 | 0 | 0 |
|  | 2 < D(X, Y) <= 3 | 3 | 0 | 0 | 0 | 0 |
|  | D(X, Y) > 3 | 4 | 0 | 0 | 0 | 3 |

|  |  | Comp | | | | |
| --- | --- | --- | --- | --- | --- | --- |
|  |  | Exact | 0 < D(X, Y)  <= 1 | 1 < D(X, Y)  <= 2 | 2 < D(X, Y)  <= 3 | D(X, Y)  > 3 |
| Coder  A | Exact | 55 | 8 | 2 | 2 | 4 |
|  | 0 < D(X, Y) <= 1 | 4 | 1 | 2 | 1 | 0 |
|  | 1 < D(X, Y) <= 2 | 0 | 3 | 2 | 1 | 1 |
|  | 2 < D(X, Y) <= 3 | 0 | 2 | 0 | 1 | 0 |
|  | D(X, Y) > 3 | 0 | 0 | 0 | 0 | 7 |

|  |  | Comp | | | | |
| --- | --- | --- | --- | --- | --- | --- |
|  |  | Exact | 0 < D(X, Y)  <= 1 | 1 < D(X, Y)  <= 2 | 2 < D(X, Y)  <= 3 | D(X, Y)  > 3 |
| Coder  B | Exact | 56 | 8 | 5 | 3 | 7 |
|  | 0 < D(X, Y) <= 1 | 3 | 1 | 1 | 2 | 0 |
|  | 1 < D(X, Y) <= 2 | 0 | 4 | 1 | 0 | 1 |
|  | 2 < D(X, Y) <= 3 | 0 | 1 | 0 | 0 | 0 |
|  | D(X, Y) > 3 | 0 | 0 | 0 | 0 | 4 |

The numbers on the diagonals show the similarity of the resultant codes (i.e. agreement). While in case of human coders the majority of cases are indeed on the diagonal (which can be interpreted as that there is agreement between the coders on which examples are “easy” (Exact) or “difficult” (distances over 3 from the Gold Standard)), there are several cases (the Exact column and the Exact row) of disagreement, where one of the coders have provided the correct Gold Standard code, whereas the other provided codes that are even more than 3 edges away (5 cases in total). In three instances, both human coders provided a code that was more than 3 steps away from the Gold Standard.

In the case of the agreement between human coders and the software, in almost 60% of the examples (55 out of 97) for which a human coder provided the exact match to the gold standard, the software also provided the exact match. On the other hand, for almost all cases where the software provided an exact match to the gold standard, so did the human coders, indicating that the cases that software found “easy” were also “easy” for the human coders. Similarly to the cases between human coders, there were cases (4 and 7 respectively for coder A and B) where the software provided a code that was more than three edges away (D(X, Y) > 3) from the gold standard, despite the fact that the human coder provided the exact match to the gold standard.

We have also compared the agreement of qualitative labels assigned to each free-text diagnosis, compared to the Gold Standard. Table 13 gives the similarity matrices similar to those presented in Table 12, but with qualitative labels. As before, the values on the diagonals represent agreements.

Table 13: Similarity matrices with qualitative labels as compared to the Gold Standard.
The tables show the numbers of cases rather than percentages.

|  |  | Coder B | | |
| --- | --- | --- | --- | --- |
|  |  | Good | Acceptable | Not acceptable |
| Coder A | Good | 80 | 3 | 2 |
|  | Acceptable | 7 | 7 | 1 |
|  | Not acceptable | 2 | 0 | 0 |

|  |  | Comp | | |
| --- | --- | --- | --- | --- |
|  |  | Good | Acceptable | Not acceptable |
| Coder A | Good | 70 | 8 | 7 |
|  | Acceptable | 5 | 6 | 4 |
|  | Not acceptable | 1 | 0 | 1 |

|  |  | Comp | | |
| --- | --- | --- | --- | --- |
|  |  | Good | Acceptable | Not acceptable |
| Coder B | Good | 70 | 10 | 9 |
|  | Acceptable | 6 | 3 | 1 |
|  | Not acceptable | 0 | 1 | 2 |

The codes assigned by the human coders agree with the Gold Standard in most instances (85% (87/102) of cases). Still, there are few cases (4 in total) where one of the coders provided a Good code whereas the other coder provided a Not acceptable code for the same textual description. When compared to the software, there is a larger discrepancy between the labels assigned to codes from the human and software coders. Still, on average in 75% (76 / 102) cases, the codings provided by both the human coder and the software were of the same quality according to the panel’s qualitative assessment. In 8 cases on average (7 and 9 for coders A and B respectively), the code provided by the human coder was considered Good but the software struggled to capture clinical intent (Not acceptable). Conversely, there was a single example for which a code assigned by a human coder received a rating of “Not acceptable”, while the software coding was rated as “Good”. In 1-2 cases, both the human and software coders provided Not acceptable codes for a given textual description.

## A.5 Coding guidelines

# Guidelines for manual SNOMED CT coding of free-text diagnoses

**1) Task Overview**

The coding task involves the assignment of one or more SNOMED CT identifiers (SCTIDs) to a given free-text diagnosis. We will code diagnoses that have been noted/listed in a clinical letter under a *Diagnoses* heading, rather than coding diseases in real-time settings (e.g. during a consultation). Thus, the coding task involves some interpretation of the clinical intent expressed by a free-text diagnosis expression. As a coder, you will use your clinical judgement to find the code(s) for the most suitable concept(s) that reflect the likely clinical intent in a particular diagnosis.

We concentrate only clinical findings i.e. disorders (including problems, diseases). All assigned (core) clinical concepts should therefore be of type *Disease (disorder)* (in addition to any qualifiers). Note that in this exercise we will not code other SNOMED CT concept types, e.g. *procedures*, *situations, social context* etc.

# 2) General coding strategies – what and how to code

**A. Code disorders specified in a free-text diagnosis**

The main task is to identify and code clinically relevant disorders/diseases/problems that are explicitly mentioned in a free-text diagnosis. While we aim to code likely clinical intent, we do not want to infer (any additional, not explicitly mentioned) disorders from free-text expressions. Still, we will aim to code as much of the context as possible, in particular if there is an existing, pre-coordinated SNOMED CT concept that corresponds to the stated disorder.

If a free-text diagnosis explicitly mentions a clinical procedure, we can code it but it will not be used in the analyses. Again, do not infer a problem based on the nature of a stated procedure, but if the problem is explicitly stated in the wording of the procedure, then code the problem. For example, in "*cataract surgery*", we will code "*cataract*" as a problem, and optionally *cataract surgery*" as procedure. However, don't make assumptions: "*CABG*" would only be coded as a procedure and we should not infer that there is underlying CAD as a problem; similarly, "*appendectomy*" would only be coded as a procedure and not also as "appendicitis".

**Note 1:**

Please also note that in metonymic cases such as, for example, when the name of a virus is used to describe the associated disorder, it is important to refine the search query in order to retrieve the SCT code for the associated disorder rather than just record the code for the virus (which will probably be the code of an organism).

**Example: ‘E. Coli’ should not be coded with a code for an organism.**

**B. Pre-coordination**

For many health conditions and procedures, there are pre-defined SNOMED CT concepts, which fully present quite specific health states, diagnoses or interventions. For example, there is a single code for “*Seropositive errosive rheumatoid arthritis”* (SCTID: 308143008). In this case a **single,** **predefined** SNOMED CT concept that can be used to describe the meaning of the diagnosis. This coding strategy is called **pre-coordination**, and it is the **preferred** way to code diagnoses: *whenever possible, select a single concept to code a given diagnosis or procedure*.

**Example:** “*sever asthma*” should be mapped to *Severe asthma (disorder) SCTID: 370221004,* rather than mapping it to two separate concepts: “*severe*” SCTID 24484000 and “Asthma (disorder)” SCTID: 195967001.

**Note 2:** Although pre-coordination and post-coordination (see below) can result in equivalent semantic descriptions for the same diagnosis, pre-coordination is the preferred coding option for this task and it should be explored before post-coordination. The pre-coordinated terms should be of type *disorder* as only these codes will be used for analyses.

**C. Post-coordination**

There might be cases where there is not a single concept that describes a given diagnosis. In that case, we can **combine** two or more concepts. This strategy is called **post-coordination**. Note that one of these codes has to be of the disorder type (which will be used for analyses), but other codes can be used for clarity and/or completeness.

**Example:** There is not a single code to represent “*severe headache*”; instead, we need to use two codes, one to represent “headache” (SCTID 25064002) and a separate code for the modifier “*severe*” (SCTID 24484000). These two codes are then placed together as post-coordination combination of codes 24484000 and 25064002.

When coding a diagnosis for which there is not a single pre-coordinated concept that captures all the aspect of a given diagnosis, we should identify *modifiers* to the core concept, including:

- Locus/finding site e.g. “liver disease”
- Laterality e.g. “*right* eye infection”
- Severity e.g. “*severe headache*”
- Chronicity/temporal associations e.g. “post-viral disorder”
- Finding method e.g. “lung cancer detected by biopsy”
- Causative associations e.g. “pancreatitis due to infection”

and then aim to establish whether there is a concept that captures at least some of these modifiers together with the core concept in a pre-coordinated concept. It is suggested that the modifiers are checked in the order specified above (i.e. give priority to body structure/laterality, then chronicity and then severity). A good strategy is to look at the hierarchy starting from the core disease term and see if any additional modifier has been already pre-coordinated.

**Example:** *“Mild right sacroiliitis”* should be coded by post-coordinating

- Inflammation of sacroiliac joint (disorder); SCTID: 55146009
- Right (qualifier value); SCTID: 24028007
- Mild (qualifier value); SCTID: 255604002

Note that we prefer using “Right (qualifier value)” instead of “Structure of right sacroiliac joint (body structure); SCTID: 722778007” to avoid repeating information (given that “Inflammation of sacroiliac joint” already include the information about the body part).

**Example:** *“fractured right arm”* is made up of the core concept “*arm fracture*” and laterality qualifier “*right*”:

- Fracture of upper limb (disorder); SCTID: 23406007
- Right (qualifier value); SCTID: 24028007

So, the coding here is a post-coordination of two SCTIDs: 23406007+24028007.

An alternative (but not preferred) coding is to consider the core concept “*fracture*” and laterality indicator “*right arm*”:

- - Fracture of bone (disorder) SCTID: 125605004
  - Right upper arm structure (body structure) SCTID: 368209003

However, note that “Right upper arm structure (body structure)” may refer to “upper arm”, which might be misleading.

**Example:** “*Cataract surgery (right eye)*” should be coded as

- Cataract (disorder); SCTID: 193570009
- Right (qualifier value); SCTID: 24028007

Note also that qualifier *right* cannot be combined with a procedure, so if the procedure is coded, then it should be coded as:

- Cataract surgery (procedure); SCTID: 110473004
- Right eye structure (body structure); SCTID: 18944008

rather than

- Cataract surgery (procedure); SCTID: 110473004
- Right (qualifier value); SCTID: 24028007 [procedure can’t be right or left]

**Example:**  Temporal context should be encoded using a relevant qualifier; for example, “*Previous pulmonary embolism*” should be coded as

- Pulmonary embolism (disorder); SCTID: 59282003
- Previous (qualifier value); SCTID: 9130008

**Example:** Suspected diagnoses should be coded using “Probable diagnosis (contextual qualifier) (qualifier value); SCTID: 2931005” if there is not an appropriate pre-coordinated term. For example, **“***Likely primary Raynaud’s*” should be coded as

- Isolated primary Raynaud's phenomenon (disorder); SCTID: 361131008
- Probable diagnosis (contextual qualifier) (qualifier value); SCTID: 2931005

**Note 3:** As a rule, in the case of a post-coordinated expression, find first the SCTID of the core disease/disorder concept, followed by the SCITDs of concepts that are used to supplement, refine or modify the meaning of the core disease concept. Use a combination that has a minimal number of concepts and avoids duplication of information.

**D. Distinct clinical concepts**

When two or more **distinct** clinical concepts are present in the same narrative description, these should be coded as separate concept. Special care should be taken not to confuse the situation with that of post-coordination. A typical example is a disorder and an associated procedure, which should be coded separately as two annotation concepts.

**Example:** “*Anxiety and Depression*” make up two different concepts that should be mapped to separate SNOMED concepts:

- Anxiety disorder (disorder); SCTID: 197480006
- Depressive disorder (disorder) SCTID: 35489007

These two concepts are not post-coordinated: there are two separate codes for two diagnoses. Clinical judgement should be used to establish that a given description is about two (or more) conditions, rather than one.

In cases where there is a pre-coordinated term that combines disorders (see below for examples), we will prefer the pre-coordinated term.

**Example: “***Prior MI and stents”* should be coded as

- Myocardial infarction (disorder); SCTID: 22298006
- Prior diagnosis (contextual qualifier) (qualifier value); SCTID: 48318009

and additionally *‘stents’* as a procedure:

- Insertion of arterial stent (procedure); SCTID: 233404000

**Example:** “*Pancreatitis due to gallstone*” should be coded as a single pre-coordinated diagnosis (“Gallstone pancreatitis (disorder); SCTID: 95563007”), rather than “Pancreatitis (disorder); SCTID: 75694006” and “Gallbladder calculus (disorder); SCTID: 235919008”. Similarly, *“CKD stage 1 due to hypertension”* should be coded as one concept: “Chronic kidney disease stage 1 due to hypertension (disorder); SCTID: 117681000119102”.

**Note 4:** The rules for pre/post-coordination would still apply for each separate concept.

**Note 5**: In cases when the same concept is repeated in the same clinical description (for example, when a clarification is given within a parenthetical expression), just record the associated SCT code only once.

**Note 6:** In cases of multiple concepts which are synonymous to each other just record the code for one of them (preference should be given to disorder type and then to the most specific concept in the hierarchy). Do not use OR or AND Boolean operators to signify synonymy or conjuction of concepts.

**E. Parent vs. child**

If there is uncertainty about choosing between a more general (i.e. parent concept) and a more specific concept (i.e. child), go for the more specific concept if applicable. If candidate SNOMED CT concepts are children of the same parent, use the SCTID of the parent concept. For example, if there is uncertainty about choosing among 'rheumatic arteritis' and 'senile arteritis', then use the SCTID of the parent concept 'arteritis'.

**3) General guidelines – what and how *not* to code**

**F. Do not code diagnoses as situations**

SNOMED CT provides *Situations* as a type of (pre-coordinated) concept that specifically includes a definition of the context of use of a clinical finding or procedure. We will not use situations for coding; rather whenever we have a disorder (procedure), code it as a *Disorder* (or *Procedure*) and post-coordinate if necessary with relevant qualifiers. The main reason for this is practical: the aim of our exercise is to evaluate coding of diagnoses (and not situations).

**Example:** History or past diagnosis should be coded using a relevant disorder term and a suitable qualifier, even when there is a pre-coordinated situation. For example, “*History of hypertension*” should be coded as

- Hypertensive disorder, systemic arterial (**disorder**); SCTID: 38341003
- History of (contextual qualifier) (qualifier value); SCTID: 39252100

rather than “History of hypertension (**situation**); SCTID: 161501007”. Similarly, “Past hypertension” should be coded as

- Hypertensive disorder, systemic arterial (disorder); SCTID: 38341003
- In the past (qualifier value); SCTID: 410513005

Note that there isn’t a pre-coordinated term here. Also, use lexically closest qualifier to the one that appeared in the free-text diagnosis as long as it satisfies the clinical intent (so, if ‘*past’* appears in the description, use it rather than ‘*history of*’ to find a suitable qualifier).

**G. Do not post-coordinate specific finding/diagnostic methods with diagnoses**

If specific finding methods and measurements are mentioned as part of diagnosis descriptions, do not post-coordinate them with the main diagnoses. However, if there is a pre-coordinated concept that captures the whole description, use it as more appropriate.

**Example:** “*Chronic renal impairment (eGFR 44)”* should be coded as

*Chronic kidney disease (disorder) SCTID: 709044004*

Note that there is a code for “*eGFR (observable entity)* SCTID: 80274001”; while we could post-coordinate the diagnosis (SCTID: 709044004) with the finding method (eGFR is a Finding Method i.e. a permissible attribute for a clinical finding), we will not code that in this exercise. However, *“Chronic renal impairment (stage 1)”* should be coded as “Chronic kidney disease stage 1 (disorder); SCTID: 431855005” Similar examples (code only the underlined parts):

*Osteopenia on DEXA – 2012*

*Osteoporosis – DEXA 2014 T score -3.2 spine, -3.7 femur*

*Mild right sacroiliitis (from previous MR scan)*

Still, use judgement to code any finding/diagnostics that has clinical significance (e.g. of a particular diagnostic value would indicate a necessary severity modifier).

**H. Do not post-coordinate drugs/treatments associated with a disease**

In this task we will not code drugs or treatments unless they have been pre-coordinated as a disorder term.

**Example:** “*Atrial fibrillation (on Warfarin)*” we should not code “*Warfarin*”, rather only

- Atrial fibrillation (disorder); SCTID: 49436004

**Example:** “*Treated vitamin D deficiency 2012”* should be coded only as

- “Vitamin D deficiency (disorder); SCTID: 34713006”

**Example:** We will ignore the context about drugs or treatments unless there is a pre-coordinated disease term. For example, “*Hypertension caused by contraceptive pill”* should be coded as

- “Hypertension caused by oral contraceptive pill (disorder); SCTID: 169465000”.

**I. Do not code *explicit* *temporal* context (e.g. dates)**

Do not code explicit temporal information (e.g. dates of diagnoses or procedures), but code qualifiers such as *recent, prior, history of* (see examples above). In cases where a procedure is planned for future, do not encode such procedures at all.

**Example:** “*Right total hip replacement February 2015*” should be coded as

- Total replacement of right hip joint (procedure); SCTID: 443435007

*Note*: there is a code for *total hip replacement* (Total replacement of hip (procedure); SCTID: 52734007) as well for ‘*right hip*’ (Right hip region structure (body structure) SCTID: 287579007), but we prefer a pre-coordinated term, rather than post-coordination.

Relevant temporal context can be still coded using appropriate qualifiers (but note that these will not be used for evaluation).

**Example**: in “*Recent eye cataract surgery”*, we will code “*Recent”* as a qualifier

- Cataract surgery (procedure); SCTID: 110473004
- Recent (qualifier value); SCTID: 6493001

Note that we will also code the associated (explicit) disorder:

- Cataract (disorder); SCTID: 193570009

**J. Do not post-coordinate causative associations** (unless pre-coordinated)

**Example**: “*pancreatitis due to infection*” should be only coded as “*pancreatitis*”. However, as indicated above, “*Pancreatitis due to gallstone*” should be coded as a single diagnosis (“Gallstone pancreatitis (disorder); SCTID: 95563007”) as there is a pre-coordinated term.

**K. Do not post-coordinate information that is redundant/obvious**

**Example:** in **“***Likely primary Raynaud’s in hands and feet*”, since *Raynaud’s* can only be in hands and feet, aim to code only the rest of the diagnosis (**“***Likely primary Raynaud’s”*).

**L. Do not code *outcomes***

**Example:** in **“***Recent right tennis elbow, improved on recent injection”* – we will not code that the problem has “*improved”.* We will still however code two clinical concepts (procedure and problem), with optional temporal qualifiers (i.e. *recent* – not shown below):

- Tennis elbow injection (procedure); SCTID: 274496007
- Right elbow region structure (body structure); SCTID: 368149001
  [Note: We cannot use Right (qualifier value); SCTID: 24028007 as procedures can’t be right or left.]

and

- Lateral epicondylitis (disorder); SCTID: 202855006.
- Right (qualifier value); SCTID: 24028007

**4) Summary: finding the right code(s) for a diagnosis free-text description**

**Main principles:**

1. The main task is to code free-text **diagnoses**. Capture as much as possible in a single pre-coordinated disorder term. If free-text explicitly refers to a **procedure**, code it as a separate concept if needed for completeness and clarity.
2. Explore the possibility of a pre-coordinated term for the whole description, ignoring the parts we do not want to encode.
3. Identify if a single disorder is expressed in a free-text diagnosis. If not, apply the steps to separate core diagnoses (and procedures).

**Steps:**

**Step 0:** Check if the free-text description contains any clinically relevant concept; this is to save time and avoid errors because in some case there is just a term like *'weight'* or *'history'* in the data.
**Step 1:** Check pre-coordination first; this is the preferred option and should be checked before anything else, even if there is suspicion about post-coordination in the description.

If the above steps fail, use synonyms, abbreviations or parent terms for the core concept (see also tips below).

**Step 2:** Checking for multiple (distinct) concepts as discussed in the guidelines; the point here is to decompose the term and search for pre-coordination for each concept separately again (before moving to post-coordination for each of them if needed).

**Step 3:** Post-coordinate if needed; since post-coordination involves checking for codes of the core concepts and its qualifiers separately, you can apply Step 1 (i.e. similar to doing pre-coordination for core concept only, pre-coordination for a qualifier only etc.).

**Tips:**

- Try synonyms: It is likely that a clinical term is expressed as a synonym, abbreviation or even a ‘lay term’ of a SNOMED CT concept. For example, in the area of rheumatology, ‘Osteonecrosis’ may be used as a synonym of ‘Avascular Necrosis’ and ‘CPDD’ an abbreviation of ‘Calcium Pyrophosphate Dihydrate Crystal Deposition Disease’.
- Try longer forms of the term – quite often, they are described as synonyms of an existing SNOMED CT concept and the browser might match that.

**Example:** ‘CFIDS’ may return nothing, but ‘Chronic Fatigue’ might give matches

- If nothing is returned, try stripping plurals, modifiers (*left*, *right*, etc.), or try with just a couple of letters and then browse the hierarchy to find a good match.
- Terms that include words such as: “*and*”, “*or*”, “*with*” etc. may contain multiple concepts and may need to be searched separately; search each term by breaking it up into core concept (e.g., head noun) and other term components.

1. Note that *disorder* codes fall beneath the *Clinical Finding* hierarchy of SNOMED CT. [↑](#footnote-ref-1)
